# Dynamic zero-COVID policy and healthcare utilization patterns in China during the Shanghai COVID-19 Omicron outbreak

**DOI:** 10.1038/s43856-023-00375-w

**Published:** 2023-10-11

**Authors:** Hong Xiao, Fang Liu, Joseph M. Unger

**Affiliations:** 1 Public Health Sciences Division, Fred Hutchinson Cancer Center, Seattle, WA USA; 2 Independent Researcher, Beijing, China

**Keywords:** Health services, Public health

## Abstract

**Background:**

In April 2022, an outbreak of the SARS-CoV-2 virus Omicron variant in Shanghai precipitated an extensive lockdown. We assessed changes in healthcare utilization during this outbreak and investigated the relationship between the stringency of mitigation strategies and disruptions in healthcare utilization.

**Methods:**

Using provincial-level data from routine health information systems covering all hospitals across Mainland China, we conducted an interrupted time series analysis to examine changes in healthcare utilization during the Shanghai outbreak. Linear regression was used to evaluate the direction and magnitude of the association between the relative changes in the move-out movement index, a proxy for the stringency in population-level mitigation strategies, and the estimated relative changes in healthcare utilization.

**Results:**

Overall, there were 22.9 billion outpatient visits and 1.2 billion discharged inpatients during the study period from January 2016 to May 2022, including 9.1 billion (39.7%) and 0.46 billion (38.2%) in the post-COVID-19 period (January 2020–May 2022), respectively. From March through May 2022, the outbreak resulted in an accumulative loss of 23.5 million (47%) outpatient visits and 0.6 million (55%) discharged inpatients in Shanghai, and a loss of 150.3 million (14%) outpatient visits and 3.6 million (7%) discharged inpatients in other regions. We find that for every 10-percentage point reduction in the relative change of move-out index, a 2.7 (95% CI: 2.0–3.4) percentage point decline in the relative change of outpatient visits, and a 4.3 (95% CI: 3.5–5.2) percentage points decline in the relative change of inpatient discharges.

**Conclusions:**

The Shanghai COVID-19 Omicron outbreak associates with a substantial reduction in outpatient visits and inpatient discharges within Shanghai and other regions in China. The stringency of the COVID-19 lockdown policies associates with more profound reductions in healthcare utilization.

## Plain language summary

In April 2022, outbreaks in Shanghai owing to the SARS-CoV-2 virus Omicron variant prompted China’s most extensive lockdown since the 2020 Wuhan shutdown. We use mathematical models to study the impact of stringent lockdown measures on the nation’s use of healthcare services. We estimate that Omicron outbreak have reduced the number of outpatient visits and inpatients discharged from hospitals by 47% and 55%, respectively, in Shanghai alone, and 14% and 7% at the nationwide level. We show there is a direct correlation between increased level of strictness of lockdown strategies and reduction in healthcare usage. Our findings highlight the importance of investing in preparations of future pandemics, so that access to essential healthcare facilities is unaffected

## Abstract (web summary)

Xiao et al. evaluate the impact of China’s Dynamic zero-COVID policy during the Omicron outbreak on the nation’s healthcare utilization. Aside from regional COVID-19 severity and health system performance, the stringent lockdown policy drastically reduces access to inpatient and outpatient services.

## Introduction

The first cases of the COVID-19 pandemic were reported in Wuhan, China, in late 2019^1^. Owing to strict containment measures and a centralized epidemic response system, the initial wave of the pandemic in China was quickly controlled^2–4^. Since March 2020, reported COVID-19 incidence and mortality in China have remained at very low levels compared to most countries^5^. Locally transmitted COVID-19 infections in China occurred after containment measures were relaxed. However, these outbreaks have been minor, even while other regions in the world continued to struggle to control repeated waves of COVID-19^2,6^. The Delta variant of the SARS-CoV-2 virus, first identified in India in late 2020^7^, quickly became the dominant strain worldwide, causing an expansive wave of new infections^8^. In response to the spread of this highly transmissible variant, China adopted a “Dynamic zero-COVID” policy, which mandated that cases of infection be kept at or near zero using a strict and timely trace and manage approach (to “find one, end one”), sometimes accompanied by lockdown mandates^6^.

In March 2022, COVID-19 cases in China hit a 2-year high owing to an outbreak of the Omicron variant in Shanghai^9^. To reduce the spread of the pandemic, Shanghai announced a phased lockdown in late March followed by a city-wide lockdown beginning on April 3. Mitigation measures included movement restrictions, home confinement, and suspension of all non-essential production and commercial activities^9,10^. The lockdown in Shanghai—China’s most populous city—represented the most extensive lockdown since the Wuhan shutdown in the initial phase of the pandemic in 2020. Despite the efficacy of the lockdown in Shanghai in ultimately stopping widespread virus transmission, it nonetheless produced a broad range of secondary social, economic, and health consequences^9,11,12^. In addition, at least 71 cities in 21 provinces reported COVID-19 cases related to the Shanghai outbreak by the end of March 2022^13^. In pursuit of zero-COVID, localized COVID-19 lockdowns proliferated across China; as of the second week of April 2022, this has led to full or partial lockdowns in 45 cities (with a combined 373 million people) that accounted for 40% of China’s economic output^14^.

Studies that assess COVID-19-induced changes in healthcare utilization in China have only assessed changes in healthcare utilization in the first few months of the pandemic or have covered small geographic areas or a limited number of health facilities^15–18^. Thus, little is known about the susceptibility and resilience of healthcare services at local or national levels during successive waves of COVID-19 under the Dynamic Zero-COVID policy. In this study, we examine changes in patterns of healthcare utilization in Shanghai and other regions in China during the Shanghai COVID-19 Omicron Outbreak in 2022. We also explore the relationship between the stringency of the COVID-19 containment response and the magnitude of the disruptions in healthcare utilization. We estimate that Omicron outbreaks reduce  the number of outpatient visits and inpatients discharged from their hospital stay by 47% and 55%, respectively, in Shanghai alone, and 14% and 7% at the nationwide level. We show that the increased level of stringency of regional and local lockdown measures is strongly related to the reduction in healthcare utilization.

## Methods

### Data sources and outcomes

Healthcare service utilization data were from the Center for Health Statistics and Information, National Health Commission of China. Healthcare service utilization volumes in both public and private hospitals at all levels (i.e., primary, secondary, tertiary hospitals, and unclassified hospitals) are required to be reported directly on a monthly basis to the provincial-level electronic direct-reporting platforms. Provincial Health Commissions verify and review the data at the primary level, investigate missing reports, and report monthly aggregates to the National Health Commission. The National Health Commission provides technical training and support to staff at the provincial and county level involved in the reporting process, and oversees and checks the quality of data at the secondary level. The monthly number of hospital visits (outpatient and emergency department visits) and number of inpatients discharged from hospitals between January 2016 and May 2022 by region (province, municipality, and autonomous region) in mainland China (except Hong Kong and Macau) were obtained. Yearly population estimates by region were extracted from China Statistical Yearbooks. Daily counts of COVID-19 cases by province were obtained from the COVID-19 Data Repository provided by the Center for Systems Science and Engineering at Johns Hopkins University^19^.

### Variable definitions

The primary outcomes were aggregate, province-level monthly number of hospital visits (outpatient and emergency department visits) and number of inpatients discharged from hospitals. The move-out movement index, a mobility index sourced from the Baidu Migration Index (BDMI), was used to represent how extensive were local lock-down measures and served as a proxy for the stringency index (with low mobility indicating more stringent restrictions)^20,21^. The BDMI is the most widely used mobility data in China. It is derived based on travel data from over 120 billion daily location requests from mobile phone apps, including Baidu Map that uses Baidu’s location services^20^. The move-out movement index of a specific area (city or province) is measured as the daily proportion of users that travel out of this region. The BDMI also provides other indices at different spatial scales, including a measure of within-city mobility intensity and one of inter/intra-provinces mobility intensity. These latter indices were not used since Baidu stopped updating them after May 2020^20^. As the move-out movement index for each provincial capital reflects both the inter- and intra-provinces mobility intensity, we used it as the proxy for the average stringency level in each province.

The analysis of de-identified, publicly available summary data does not constitute human subjects research as defined by regulation (45 CFR 46.102[d]). Therefore, no additional ethical approval was sought for conducting the study from authors’ affiliated institutions.

### Statistics and reproducibility

We used an interrupted time series analysis^22^ to estimate changes in healthcare utilization associated with the Shanghai COVID-19 Omicron outbreak beginning in March 2022. Given a disperse variation structure in monthly counts of utilization, we modeled the outcomes using a segmented negative binomial regression parameterization defining both pre-COVID-19 trends (January 2016 to December 2019) and distinct post-COVID-19 periods that reflected different pandemic periods as experienced within China. We included a linear effect of time to capture the long-term secular trend of healthcare utilization in the pre-COVID period. We also included fixed-effect monthly indicators and the number of spring festival days to account for observed seasonal patterns and the effect of the spring festival. The negative binomial model equation (Eq. 1) estimating monthly utilization was expressed as follows:

1 $${{{{{\rm{E}}}}}}({{{{\mathrm{ln}}}}}({Y}_{t}))= 	 \,{\beta }_{0}+{\beta }_{1}{{{{{{\rm{T}}}}}}}_{t}+{\beta }_{2}\,{{{{{\rm{Lockdown}}}}}}+{\beta }_{rec1}({{{{{{\rm{T}}}}}}}_{{{{{{\rm{t}}}}}}}-{{{{{{\rm{T}}}}}}}_{{{{{{\rm{t}}}}}}1})\\ 	 +{\beta }_{rec2}({T}_{t}-{T}_{t2}) +\mathop{\sum }\limits_{i=3}^{5}{\beta }_{SHi}{Omicro}{{n}}_{ti}+\mathop{\sum }\limits_{j=2}^{12}{\beta }_{mj}{M}_{tj}\\ 	 +{\beta }_{3}{{{{{{\rm{SF}}}}}}}_{{{{{{\rm{t}}}}}}}+{{{{{\rm{offset}}}}}}\left(\right.{{{{\mathrm{ln}}}}}({{{{{{\rm{P}}}}}}}_{{{{{{\rm{t}}}}}}})$$

Here, Y_t_ represents healthcare utilization volume, β_0_ represents the estimated volume at the beginning of the pre-pandemic period, β_1_ represents the monthly change over the pre-COVID period, T_t_ represent the time since the start of the study period, β_2_ is the change in healthcare utilization during the initial lockdown period (February-March 2020, denoted by the indicator variable Lockdown), β_rec1_ represents the average monthly change in utilization during the initial recovery period from April to June 2020 when the first wave had been controlled and lockdowns had mostly been lifted throughout China, and β_rec2_ represents the average monthly change in utilization during the subsequent recovery period from July 2020 onward, during which time COVID-19 cases have remained at a low level nationwide. T_t_-T_t1_ and T_t_-T_t2_ represent the times from March 2020 and June 2020, respectively. *β*_*SHi*_ is the change in utilization in each month during the Shanghai Omicron outbreak, and *Omicron*_*ti*_ is the indicator of each month of the Shanghai Omicron outbreak from March to May 2022. M_tj_ is the indicator of calendar month with the month of January as the reference category, SF_t_ is the number of days of the spring festival holiday, and P_t_ represents the catchment population of hospitals in each province. Newey-West standard errors with autocorrelation of up to three lags were used.

We reported the incidence rate ratio (IRR) of estimates for model-fitted (factual) versus model-expected (counterfactual; i.e., had the Shanghai outbreak not occurred) estimates, quantified as exp(${\beta }_{SHi}$), for each month from March to May 2022. Secondarily, we estimated the overall change, calculated as the difference between the sum of fitted monthly outcomes in the presence of the Omicron outbreak (i.e., factual estimate) and that in the absence of the outbreak (i.e., counterfactual estimate), over the entire period (March to May 2022). We simulated 1000 predictions per month under each scenario (factual estimate and counterfactual estimate) using the estimated coefficients and their variance-covariance matrix of the multivariate normal distribution derived from the model. The 2.5 and 97.5 percentiles of the simulated values represented the 95% confidence interval of the difference. P-values were calculated as the smaller of the proportion of simulated values falling above or below zero (depending on the direction of the comparison), multiplied by two to indicate a 2-sided p-value.

We conducted regression analyses for outpatient visits and inpatient volume separately and repeated the analyses for Shanghai and each of the other provinces. We also used linear regression to evaluate the association between the relative changes in move-out movement index and the estimated relative changes in healthcare utilization. The change in the move-out movement index was calculated as the average relative change in the daily move-out movement index of a month compared to that of the same month in the prior year.

All analyses were conducted in R-Version 4.0.2 (R Project for Statistical Computing). A two-sided *p* < 0.05 was deemed to be statistically significant. This study is reported as per the Strengthening the Reporting of Observational Studies in Epidemiology (STROBE) guidelines for cohort studies.

### Reporting summary

Further information on research design is available in the Nature Portfolio Reporting Summary linked to this article.

## Results

The total number of hospitals in the analysis increased from 27,226 in 2016 to 36,451 in 2022, serving a total population of approximately 1.4 billion people. Significant increasing trends in monthly hospital outpatient visits and inpatient volume were observed across the pre-COVID period. These temporal trends were accounted for in the regression model. In total, there were about 22.9 billion outpatient visits and 1.2 billion discharged inpatients during the study period from January 1, 2016 to May 31, 2022, including 9.1 billion (39.7%) outpatient visits and 0.46 billion (38.2%) discharged inpatients in the post-COVID period (Table 1). A total of 224,131 COVID-19 cases were reported in the study period, including 81,752 (36.5%) before April 2020, and 27,667 (12.3%) from April 2020 to February 2022. Since the Shanghai outbreak, 114,603 (51.2%) COVID-19 cases were reported among all provinces, municipalities, and autonomous regions except Tibet and Ningxia, ranging from 12 in Xinjiang to 58,579 in Shanghai (median: 457; Interquartile Range: 737) (Table 2).

**Table 1:** Number of outpatient visits and inpatient discharges, Jan 2016—May 2022.

		2016	2017	2018	2019	2020	2021	2022 (January-May)	2022 (March-May)
Shanghai	Outpatient visits^a^	150,312	153,178	159,422	169,181	133,036	169,125	59,851	26,332
	Inpatients Discharged^a^	3409	3687	3997	4510	3632	4299	1158	490
Other regions	Outpatient visits^a^	3,022,489	3,204,599	3,376,381	3,554,440	3,153,946	4,014,284	1,557,986	945,411
	Inpatients Discharged^a^	165,234	178,612	191,145	197,586	178,701	194,107	80,150	49,190
Total	Outpatient visits^a^	3,172,801	3,357,777	3,535,803	3,723,621	3,286,982	4,183,409	1,617,837	912,743
	Inpatients Discharged^a^	168,643	182,299	195,142	202,096	182,333	198,406	81,308	49,680
	Number of hospitals	27,226	28,751	30,294	32,476	33,972	35,112	36,451	36,451

^a^Numbers measured in thousand.

**Table 2:** Population and number of COVID-19 cases in each region of Mainland China, Jan 2020–May 2022.

Region	Population in 2022 (million)	COVID-19 Cases							
		2020	2021	Jan-22	Feb-22	Mar-22	Apr-22	May-22	Jan-May, 2022
Shanghai	242.4	1516	1587	662	585	2104	51098	5457	59906
Anhui	632.4	993	16	0	3	25	28	0	56
Beijing	215.4	987	224	137	131	290	386	1227	2171
Chongqing	310.2	590	21	2	7	71	7	14	101
Fujian	394.1	513	850	92	107	1215	242	243	1899
Gansu	263.7	182	174	0	12	313	0	0	325
Guangdong	1134.6	2046	1411	304	924	1907	462	249	3846
Guangxi	492.6	264	358	93	400	364	108	47	1012
Guizhou	360	147	13	1	1	12	5	6	25
Hainan	93.4	171	19	0	1	5	92	0	98
Hebei	755.6	373	1085	15	3	476	46	7	547
Heilongjiang	377.3	964	1071	29	19	323	546	32	949
Henan	960.5	1299	342	1015	11	162	77	276	1541
Hubei	591.7	68149	168	3	37	34	7	1	82
Hunan	689.9	1021	201	8	3	91	61	8	171
Jiangsu	805.1	684	941	5	149	232	195	29	610
Jiangxi	464.8	935	24	0	0	77	333	14	424
Jilin	270.4	157	432	2	14	28458	11148	82	39704
Liaoning	435.9	351	444	17	215	555	63	30	880
Inner Mongolia	253.4	364	822	5	407	85	67	3	567
Ningxia	68.8	75	47	0	0	0	0	0	0
Qinghai	60.3	18	12	0	0	2	63	52	117
Shaanxi	386.4	507	1712	607	0	395	56	6	1064
Shandong	1004.7	862	181	32	57	1444	141	18	1692
Shanxi	371.8	224	42	2	16	26	108	2	154
Sichuan	834.1	853	468	53	88	290	297	283	1011
Tianjin	156	309	280	461	76	661	15	172	1385
Tibet	34.4	1	0	0	0	0	0	0	0
Xinjiang	248.7	980	1	15	0	1	8	3	27
Yunnan	483	229	1588	64	75	110	53	30	332
Zhejiang	573.7	1306	710	202	47	417	427	28	1121
Total	13965.3	87070	15244	3826	3388	40145	66139	8319	121817

### Model-based estimates of change in healthcare utilization after the Shanghai Omicron Outbreak

Immediately after the onset of the outbreak in Shanghai in March 2022, there was a statistically significant decrease in both outpatient visits (IRR = 0.81, 95% CI: 0.73–0.90; *p* < 0.0001) and inpatient discharges (IRR = 0.70, 95% CI: 0.66–0.74; *P* < 0.0001) in Shanghai (Table 3). A statistically significant decrease in outpatient visits (IRR = 0.86, 95% CI: 0.82–0.89; *p* < 0.0001) was also observed in other provinces of China in March, but not in inpatient visits (IRR = 1.01, 95% CI:0.97–1.04; *p* = 0.72). Following the city-wide lockdown in Shanghai in April 2022, a more precipitous decline in healthcare utilization was observed, with a 71% (IRR = 0.29, 95% CI: 0.26–0.31; *p* < 0.0001) and 80% (IRR = 0.20, 95% CI: 0.19–0.22; *p* < 0.0001) decrease in outpatient and inpatient volume, respectively, in Shanghai, with corresponding decreases of 22% (IRR = 0.78, 95% CI: 0.75–0.82; *p* < 0.0001) and 14% (IRR = 0.86, 95% CI: 0.83–0.90; *p* < 0.0001), respectively, in other provinces. Similar, though somewhat less extreme, patterns were observed for May, 2022. During the entire 3-month period March–May, 2022, decreases in outpatient and inpatient volume were statistically significant (*p* < 0.0001) in Shanghai and the rest of China.

**Table 3:** Model-based estimates of changes in healthcare utilization

	Shanghai				Other Regions				No. Regions^a^ with Declining Utilization, *N* = 31	
	Outpatient		Inpatient		Outpatient		Inpatient		Outpatient	Inpatient
	IRR (95% CI)	*p*-value	IRR (95% CI)	*p*-value	IRR (95% CI)	*p*-value	IRR (95% CI)	*p*-value	Any Decline (Statistically Significant Decline)	
Mar-22	0.81 (0.73, 0.90)	<0.0001	0.70 (0.66, 0.74)	<0.0001	0.86 (0.82, 0.89)	<0.0001	1.01 (0.97, 1.04)	0.72	31 (28)	15 (7)
Apr-22	0.29 (0.26, 0.32)	<0.0001	0.20 (0.19, 0.22)	<0.0001	0.78 (0.75, 0.82)	<0.0001	0.86 (0.83, 0.90)	<0.0001	31 (30)	31 (23)
May-22	0.36 (0.34, 0.39)	<0.0001	0.30 (0.28, 0.31)	<0.0001	0.81 (0.78, 0.85)	<0.0001	0.92 (0.89, 0.96)	<0.0001	31 (31)	27 (18)
Overall (Mar-May)	0.53 (0.50, 0.56)	<0.0001	0.45 (0.43, 0.47)	<0.0001	0.86 (0.84, 0.88)	<0.0001	0.93 (0.90, 0.96)	<0.0001	31 (31)	29 (21)
Overall volume loss (in millions)	23.54 (21.39, 25.53)	<0.0001	0.60 (0.56, 0.65)	<0.0001	150.31 (123.45, 178.12)	<0.0001	3.56 (1.86, 5.16)	<0.0001	31 (31)	29 (21)

^a^Provinces, municipalities, and autonomous regions.

By the end of May 2022, the Omicron outbreak resulted in a cumulative loss of 23.5 million (47%, 95% CI: 44–50%; *p* < 0.0001) outpatient visits and 0.6 million (55%, 95% CI: 53–57%; *p* < 0.0001) discharged inpatients in Shanghai, and a cumulative loss of 150.3 million (14%, 95% CI: 12%–16%; *p* < 0.0001) outpatient visits and 3.6 million (7%, 95% CI: 4–10%; *p* < 0.0001) inpatient discharges in other regions. Overall, decreases were statistically significant in all regions for outpatient visits, and in 21 regions for inpatient discharges.

### Association between mobility index and healthcare utilization

**Fig. 1:**
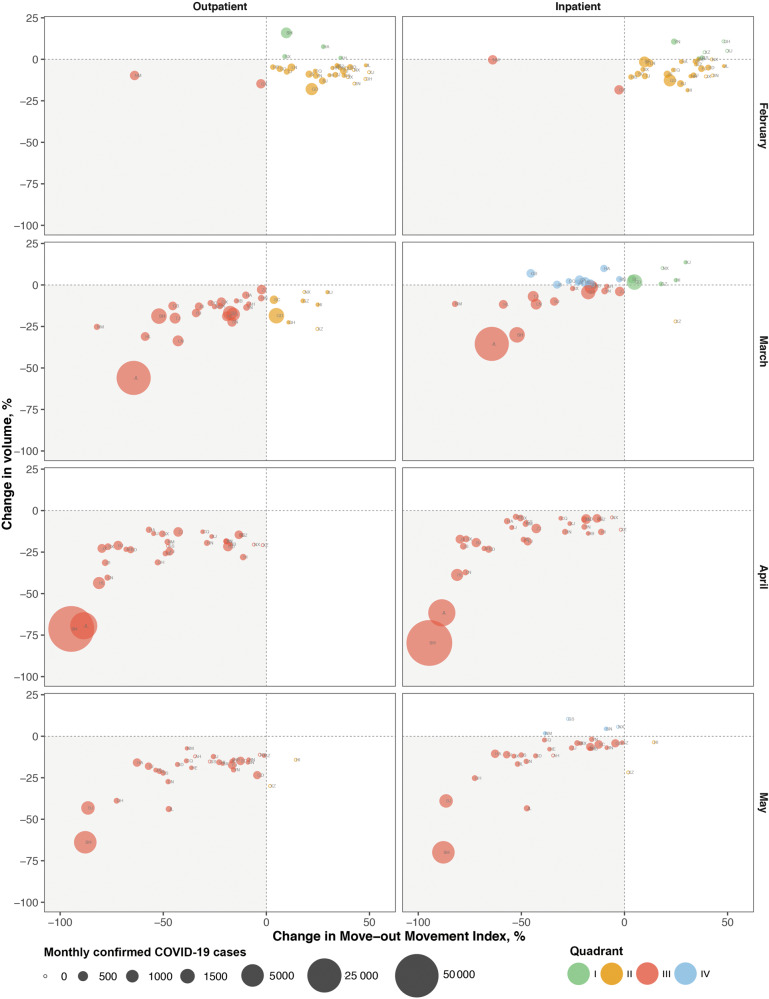
The estimated change in healthcare utilization and move-out movement index by region, February–May 2022. Quadrant I (green dots), II (yellow dots), III (red dots), and IV (blue dots) represents the upper right, bottom right, bottom left and upper left quadrant, respectively. The shading quadrant, quadrant III, denotes regions with corresponding decreases in both the move-out movement index and patient volume. The size of the is scaled proportionally to represent the number of monthly confirmed COVID-19 cases. The circles symbolize areas reporting zero monthly case.

There was a positive association between the relative change in the move-out movement index and the magnitude of change in outpatient visits or inpatient volume. For each 10-percentage point reduction in the relative change (compared to the prior year) in the move-out index (e.g., relative reduction from −50% down to −60%), a 2.7 (95% CI: 2.0–3.4; *p* < 0.0001) percentage point decline in the relative change (compared to the counterfactual estimates) of outpatient visits (e.g., relative reduction from −15% down to −17.7%), and a 4.3 (95% CI: 3.5–5.2; *p* < 0.0001) percentage points decline in the relative change of inpatient discharges, was found (Fig. 1).

**Fig. 2:**
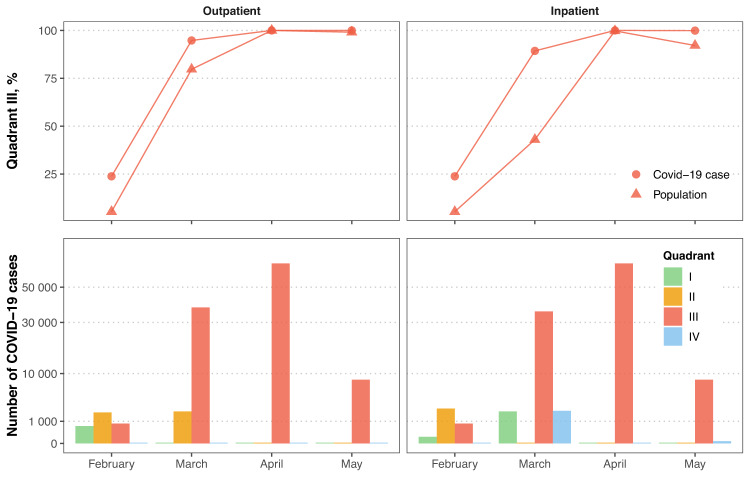
Covid-19 cases and population in regions with corresponding decreases in both the move-out movement index and outpatient/inpatient volume. Quadrant I (green bar), II (yellow bar), III (red bar), and IV (blue bar) represents the upper right, bottom right, bottom left and upper left quadrant in Fig. 1, respectively.

In February 2022, simultaneous decreases in both the move-out movement index and outpatient visits were found in two (2/31) regions that accounted for 23.8% of monthly COVID-19 cases and 5.3% of the population in China. In contrast, simultaneous decreases in both the move-out movement index and outpatient visits were found in 23 regions (94.8% of COVID-19 cases; 79.7% of population) in March 2022, and all of the 31 regions in April 2022 (Figs. 1, 2). A similar pattern was observed with respect to the move-out movement index and inpatient discharges (Figs. 1, 2).

## Discussion

This is a comprehensive examination of national patterns of healthcare utilization in China under China’s Dynamic zero-COVID policy during the Omicron outbreak centered in Shanghai. We observed dramatic reductions of more than 70% in both outpatient visits and inpatient discharges in Shanghai. Importantly, we observed notable reductions in healthcare utilization in other provinces as well. In total, nationwide in China, the magnitude of lost outpatient visits from March through May, 2022 was over 170 million, and of lost inpatient discharges was over 4 million. There was also clear evidence that the reduction in healthcare utilizations was strongly related to the level of stringency of regional and local lockdown measures.

Pressure on patients and healthcare providers, institutions, and systems imposed by the COVID-19 pandemic has been repeatedly and widely reported, both in China and worldwide^16,23,24^. Our findings regarding unmet needs in healthcare services during the lockdown in Shanghai are consistent with previously published estimates^10,25,26^. Several reasons have been cited for declining healthcare utilization during the COVID-19 pandemic. In part, the observed declines in healthcare utilization may be explained by a reduction in need. For instance, decreased mobility has led to lower incidence of road traffic injuries^27^. Reduced economic activity has been accompanied by lower levels of air pollutants and thus lower incidence of pollution-related conditions including stroke, ischemic heart diseases and chronic obstructive pulmonary diseases^27,28^. Bans on gatherings and requirements for social distancing and mandatory mask wearing have likely contributed to the reduced spread of other infectious disease including flu, infectious diarrhea, and Hand-foot-and-mouth disease^29^. However, the declines in healthcare utilization have also been shown to reflect unmet healthcare needs, due to multiple reasons including the public’s fear of COVID-19 contagion risks in health facilities, the suspension or cancellation of non-COVID-19 care, barriers imposed by lockdown policies (e.g., curfews, transport closures and home confinement), as well as financial barriers due to loss of remuneration or health insurance^15,24,30,31^. The redeployment of health workers or health facilities towards prevention and care for COVID-19 has likely also depressed care for other diseases^16,24,31^. For the Shanghai outbreak, not only were health workers from health facilities within Shanghai redeployed to provide care for COVID-19, but also, a large number of health workers from other regions were deployed to Shanghai, which likely adverse impacted healthcare utilization for regions outside Shanghai^32^.

The interruptions in care due to the COVID-19 pandemic have been demonstrated to adversely impact conditions as varied as cancer^33^, cardiovascular diseases^34^, mental health^35^, preterm birth delivery^36^, and maternal and child mortality^37^. Vulnerable populations have been disproportionately affected, including women, racial and ethnic minorities, rural residents that historically lack access to subspecialty healthcare services, those with chronic diseases, and those with low socioeconomic status^15,38–40^. There is increasing concern that persistent unmet need for healthcare services could reverse China’s decades of progress in promoting healthcare accessibility, health outcomes, and equity^24,41,42^.

A critical question for policymakers, both inside and outside of China, is how to balance population-level mitigation measures aimed at stemming the morbidity and mortality due to COVID-19 infections with the potential adverse consequences of those measures on healthcare utilization. Our findings from the Shanghai Omicron Outbreak demonstrate that strict lockdown measures can stem a COVID-19 outbreak, but also have notable collateral impacts on healthcare utilization for other diseases. The COVID-19 pandemic has proven to be far more pervasive and persistent than many first surmised, in response to which some governments have repeatedly implemented lockdowns^24,31,43^. China, for its part, has maintained the enforcement of zero-COVID strategy for more than one year. It has been argued that the unintended consequences of the COVID-19 response, including collateral impacts on public health from disruptions in the continuity of healthcare, may have outweighed the cumulative effects on morbidity and mortality from COVID-19 itself^44^. Given the resilience and persistence of the SARs-CoV-2 virus and the likelihood that waves of COVID-19 will continue to impact China, there is an urgent need to invest in strategies, both within and beyond health systems, to ensure accessibility to non-COVID-19 related healthcare, to compensate for missed services, and to prevent the exacerbation of extant unmet needs for healthcare.

Excessive epidemic control measures and the abuse of related tools in China have been repeatedly reported and criticized, particularly since the reoccurrence of COVID-19 outbreaks in Shanghai^45,46^. To address this, the central government has taken steps to attempt to optimize COVID prevention and control policies, rectify irregular virus containment practices and to refrain from making the policies rigidly uniform. On June 5 2022, the State Council’s Joint Prevention and Control listed nine practices that should be prohibited in response to growing complaints that local governments were imposing excessive travel restrictions, such as refusing to provide healthcare service for patients with acute or critical illness^47^. The National Health Commission launched a message board on June 28 2022 to receive and help address problems submitted by the public. By July 6, the commission had received nearly 14,000 complaints about excessive COVID-19 restrictions imposed by local governments, with approximately 500 complaints still received per day through the end of July^47^. These reports suggest the challenges inherent in a strategy that prioritizes reducing virus transmission over other consideration of public health, even as the recent outbreak in Shanghai has been successfully contained. Likely given these challenges, it has been reported that Chinese officials began to recalibrate its Dynamic Zero-COVID policy in November 2022, and had officially ended the Zero-COVID policy nationwide by early January 2023^46,48,49^.

Our analysis has several strengths. We estimated the change of hospital-based healthcare utilization in Shanghai and other regions of China using administrative data that represented the complete census of all hospitals in the country. The longitudinal data, spanning multiple years before and after the outbreak, provided near real-time surveillance of health system performance, and revealed a comprehensive trajectory of healthcare utilization at the sub-national level. Additionally, the corresponding move-out movement indices enabled the examination of the correlation between the stringency of COVID-19 containment response and the magnitude of the disruptions in healthcare utilization. However, our study is also subject to limitations. First, given the nature of the aggregated data, we were not able to examine healthcare utilization patterns by individual characteristics (e.g., sex, age, or disease type), nor was it feasible to examine the heterogeneity in outcomes by hospital type. We were unable to examine potential changes in the quality of healthcare or the full indirect impact on health outcomes either. Second, the data from the routine health information system generally did not include the telemedicine consultations provided through hospital or third-party platforms. Third, data on the number of tests, which could be associated with the number of confirmed cases was not available. Although some sparsely populated regions distant from the epicenter of the outbreak (such as Tibet and Ningxia) were reported to have had few or no cases, there is some uncertainty whether these low estimates are attributable to an actual absence of cases or a relatively lower number of tests conducted in these areas. Finally, given the data, we were unable to delineate those factors (e.g., changes in need or behaviors), beyond mobility restrictions, that may have also been responsible for declines in healthcare services.

This study provides a comprehensive assessment of changes of healthcare utilization in China associated with the Shanghai outbreak  to date. Nearly all regions in China, regardless of local COVID-19 severity or traditional performance of health systems, have experienced significant reductions in both outpatient visits and inpatient discharges since the Shanghai outbreak. These findings highlight the general need, both within China and beyond, to develop and design resilient healthcare systems able to anticipate, prepare for, and recover from public health emergencies, while ensuring continuity in the provision of essential healthcare service and protecting public health. Considering the prolonged nature of the COVID-19 pandemic, this study offers evidentiary benchmarks that may help guide public health researchers and policy makers as they navigate the remainder of the COVID-19 pandemic and anticipate potential future public health emergencies.

### Supplementary information

Description of Additional Supplementary Files

Summpememtary Data 1

Summpememtary Data 2

Reporting Summary

## Data Availability

Data underlying Fig. 1 could be found in Supplementary Data 1. Data underlying Fig. 2 could be found in Supplementary Data 1 and 2. The datasets generated and analyzed during the current study could be obtained from H.X. upon request.

## Code availability

The essential analysis codes used to produce the results are available in the Github https://github.com/xiaohongpku/Shanghai-COVID-19-project

## References

[1] Origin of SARS-CoV-2. https://iris.who.int/bitstream/handle/10665/332197/WHO-2019-nCoV-FAQ-Virus_origin-2020.1-eng.pdf (World Health Organization, 2020).

[2] Burki T (2020). China’s successful control of COVID-19. Lancet Infect Dis..

[3] Liu, W., Yue, X. G. & Tchounwou, P. B. Response to the COVID-19 Epidemic: The Chinese Experience and Implications for Other Countries. *Int. J. Environ. Res. Public Health* **17**10.3390/ijerph17072304 (2020). 10.3390/ijerph17072304 PMC7177503 32235413

[4] Lancet, T. (2020). COVID-19 and China: lessons and the way forward. Lancet.

[5] Wang Z, Xiao H, Lin L, Tang K, Unger JM (2022). Geographic social inequalities in information-seeking response to the COVID-19 pandemic in China: longitudinal analysis of Baidu Index. Sci. Rep..

[6] Jue L, Min L, Wannian L (2022). The dynamic COVID-zero strategy in China. China CDC Weekly.

[7] Mlcochova P (2021). SARS-CoV-2 B.1.617.2 Delta variant replication and immune evasion. Nature.

[8] Del Rio C, Malani PN, Omer SB (2021). Confronting the delta variant of SARS-CoV-2, summer 2021. JAMA..

[9] Cheshmehzangi A, Zou T, Su Z (2022). Commentary: China’s zero-COVID approach depends on Shanghai’s Outbreak Control. Front. Public Health.

[10] Chen M, Li R, Ding G, Jin C (2022). Needs of cancer patients during the SARS-CoV-2 Omicron lockdown: a population-based survey in Shanghai, China. Biosci. Trends.

[11] The L (2022). Mental health after China’s prolonged lockdowns. Lancet..

[12] Muyi, X., Isabelle, Q., Ang, L., Chang, C. A. & Vivian, W. *What Videos Show About the Extremes of China’s ‘Zero Covid’ Policy*, https://www.nytimes.com/2022/11/16/world/asia/china-zero-covid-policy-videos.html?smid=nytcore-ios-share&referringSource=articleShare (2022).

[13] Guangxi Center for Disease Control and Prevention. *Reminders from Guangxi CDC: As outbreaks emerge, please report immediately when arrivig in Guangxi**[广西疾控中心提醒: 多地疫情高发, 抵桂即刻报备]*, https://web.archive.org/web/20220402064431/http://news.gxnews.com.cn/staticpages/20220327/newgx623fbf46-20694537.shtml (2022).

[14] Wang, J. & Qi, L. *China Covid-19 Lockdowns Spread Beyond Shanghai to Other Cities*, https://www.wsj.com/articles/china-covid-19-lockdowns-spread-beyond-shanghai-to-other-cities-11649949817 (2022).

[15] Xiao H (2022). Unequal impact of the COVID-19 pandemic on paediatric cancer care: a population-based cohort study in China. Lancet Reg. Health West Pac..

[16] Xiao H (2021). The impact of the COVID-19 pandemic on health services utilization in China: time-series analyses for 2016-2020. Lancet Reg. Health West Pac..

[17] Zhang, Y. N. *et al*. Reduction in healthcare services during the COVID-19 pandemic in China. *BMJ Glob. Health* **5**. 10.1136/bmjgh-2020-003421 (2020). 10.1136/bmjgh-2020-003421 PMC7662138 33184065

[18] Dema E (2022). Initial impacts of the COVID-19 pandemic on sexual and reproductive health service use and unmet need in Britain: findings from a quasi-representative survey (Natsal-COVID). Lancet Public Health.

[19] *COVID-19* Map - *Johns Hopkins Coronavirus Resource Center*, https://coronavirus.jhu.edu/map.html (2022).

[20] Hu T (2021). Human mobility data in the COVID-19 pandemic: characteristics, applications, and challenges. Int. J. Digital Earth.

[21] Sun J, Kwek K, Li M, Shen H (2021). Effects of social mobility and stringency measures on the COVID-19 outcomes: evidence from the United States. Front. Public Health.

[22] Xiao, H., Augusto, O. & Wagenaar, B. H. Reflection on modern methods: a common error in the segmented regression parameterization of interrupted time-series analyses. *Int. J. Epidemiol.*10.1093/ije/dyaa148 (2020). 10.1093/ije/dyaa148 PMC8271192 33097937

[23] Sun S (2021). COVID-19 and healthcare system in China: challenges and progression for a sustainable future. Global Health.

[24] Arsenault C (2022). COVID-19 and resilience of healthcare systems in ten countries. Nat. Med..

[25] Li, H. et al. Impact of the COVID-19 pandemic on utilization of inpatient mental health services in Shanghai, China. *Healthcare (Basel)*. **10**10.3390/healthcare10081402 (2022). 10.3390/healthcare10081402 PMC9407850 36011058

[26] Ma, X. et al. Rapid increase in depression within the first month of the Shanghai Covid lockdown in 2022. 10.31234/osf.io/mwge8 (PsyArXiv, 2022).

[27] Liu J (2021). Excess mortality in Wuhan city and other parts of China during the three months of the COVID-19 outbreak: findings from nationwide mortality registries. BMJ.

[28] Hameed Shaikh S (2021). Low stroke admissions and mortality during COVID-19 lockdown: Link to air quality index. J. Neurol. Sci..

[29] Geng MJ (2021). Changes in notifiable infectious disease incidence in China during the COVID-19 pandemic. Nat. Commun..

[30] Unger JM, Xiao H, LeBlanc M, Hershman DL, Blanke CD (2021). Cancer clinical trial participation at the 1-year anniversary of the outbreak of the COVID-19 pandemic. JAMA Netw Open..

[31] Haldane V (2021). Health systems resilience in managing the COVID-19 pandemic: lessons from 28 countries. Nat. Med..

[32] Jiang, H. *Over 38000 health workers from 15 provinces redeployed to Shanghai during COVID-19 Omicron outbreak [15省份3.8万多名医务人员驰援上海]*, http://zj.people.com.cn/n2/2022/0405/c186327-35208619.html (2022).

[33] Luo Q (2022). Cancer incidence and mortality in Australia from 2020 to 2044 and an exploratory analysis of the potential effect of treatment delays during the COVID-19 pandemic: a statistical modelling study. Lancet Public Health.

[34] Nef HM (2021). Impact of the COVID-19 pandemic on cardiovascular mortality and catherization activity during the lockdown in central Germany: an observational study. Clin. Res. Cardiol..

[35] Boyer L (2022). Impact of the COVID-19 pandemic on non-COVID-19 hospital mortality in patients with schizophrenia: a nationwide population-based cohort study. Mol. Psychiatry.

[36] Xie Y (2022). Interrupted-time-series analysis of the immediate impact of COVID-19 mitigation measures on preterm birth in China. Nat. Commun..

[37] Ahmed T (2022). Healthcare utilization and maternal and child mortality during the COVID-19 pandemic in 18 low- and middle-income countries: an interrupted time-series analysis with mathematical modeling of administrative data. PLoS Med..

[38] Collaborators C-EM (2022). Estimating excess mortality due to the COVID-19 pandemic: a systematic analysis of COVID-19-related mortality, 2020-21. Lancet.

[39] Dang A (2022). Hospitalizations and mortality from non-SARS-CoV-2 causes among medicare beneficiaries at US Hospitals during the SARS-CoV-2 pandemic. JAMA Netw. Open.

[40] Topriceanu CC (2021). Evaluating access to health and care services during lockdown by the COVID-19 survey in five UK national longitudinal studies. BMJ Open.

[41] Yip W (2019). 10 years of healthcare reform in China: progress and gaps in Universal Health Coverage. Lancet.

[42] Wang Z, Tang K (2020). Combating COVID-19: health equity matters. Nat. Med..

[43] Myers LC, Liu VX (2022). The COVID-19 pandemic strikes again and again and again. JAMA Netw. Open.

[44] Cash R, Patel V (2020). Has COVID-19 subverted global health?. Lancet.

[45] Wang, X. *Abuse of epidemic controls criticized*, https://www.chinadaily.com.cn/a/202206/25/WS62b64afca310fd2b29e687e2.html (2022).

[46] *China to make ‘substantial’ COVID policy changes soon - ex-govt expert*, https://www.reuters.com/world/china/china-make-substantial-changes-covid-policy-soon-former-govt-expert-2022-11-04/ (2022).

[47] Wang, X. *Complaints drop around virus control*, https://www.chinadaily.com.cn/a/202207/21/WS62d8a0e8a310fd2b29e6d862.html (2022).

[48] Zhai, K. *China Weighs Gradual Zero-Covid Exit but Proceeds With Caution, Without Timeline*, https://www.wsj.com/articles/china-weighs-zero-covid-exit-but-proceeds-with-caution-and-without-timeline-11667826209 (2022).

[49] The Lancet Regional Health-Western, P. (2023). The end of zero-COVID-19 policy is not the end of COVID-19 for China. Lancet Reg. Health West Pac..

