# Supplementary material for: Dynamic zero-COVID policy and healthcare utilization patterns in China during the Shanghai COVID-19 Omicron outbreak
Source: Commun Med (Lond). 2023 Oct 11;3:143. doi: 10.1038/s43856-023-00375-w (PMC10567791; doi:10.1038/s43856-023-00375-w)
Supplement: Supplementary file 4 — Reporting Summary [file 43856_2023_375_MOESM4_ESM.pdf]

nature portfolio

Corresponding author(s): Hong Xiao, Joseph M Unger

Last updated by author(s): Aug 15, 2023

Reporting Summary

Nature Portfolio wishes to improve the reproducibility of the work that we publish. This form provides structure for consistency and transparency in reporting. For further information on Nature Portfolio policies, see our Editorial Policies and the Editorial Policy Checklist.

Statistics

For all statistical analyses, confirm that the following items are present in the figure legend, table legend, main text, or Methods section.

n/a	Confirmed	
☐	☒	The exact sample size (*n*) for each experimental group/condition, given as a discrete number and unit of measurement
☐	☒	A statement on whether measurements were taken from distinct samples or whether the same sample was measured repeatedly
☐	☒	The statistical test(s) used AND whether they are one- or two-sided<br>*Only common tests should be described solely by name; describe more complex techniques in the Methods section.*
☐	☒	A description of all covariates tested
☒	☐	A description of any assumptions or corrections, such as tests of normality and adjustment for multiple comparisons
☐	☒	A full description of the statistical parameters including central tendency (e.g. means) or other basic estimates (e.g. regression coefficient) AND variation (e.g. standard deviation) or associated estimates of uncertainty (e.g. confidence intervals)
☐	☒	For null hypothesis testing, the test statistic (e.g. *F*, *t*, *r*) with confidence intervals, effect sizes, degrees of freedom and *P* value noted<br>*Give P values as exact values whenever suitable.*
☒	☐	For Bayesian analysis, information on the choice of priors and Markov chain Monte Carlo settings
☒	☐	For hierarchical and complex designs, identification of the appropriate level for tests and full reporting of outcomes
☐	☒	Estimates of effect sizes (e.g. Cohen's *d*, Pearson's *r*), indicating how they were calculated

Our web collection on statistics for biologists contains articles on many of the points above.

Software and code

Policy information about availability of computer code

Data collection	R-Version 4.0.2 (R Project for Statistical Computing)
Data analysis	R-Version 4.0.2 (R Project for Statistical Computing)

For manuscripts utilizing custom algorithms or software that are central to the research but not yet described in published literature, software must be made available to editors and reviewers. We strongly encourage code deposition in a community repository (e.g. GitHub). See the Nature Portfolio guidelines for submitting code & software for further information.

Data

Policy information about availability of data

All manuscripts must include a data availability statement. This statement should provide the following information, where applicable:

- Accession codes, unique identifiers, or web links for publicly available datasets
- A description of any restrictions on data availability
- For clinical datasets or third party data, please ensure that the statement adheres to our policy

The datasets and codes generated and analyzed during the current study may be obtained from Dr Hong Xiao upon request.

Human research participants

Policy information about studies involving human research participants and Sex and Gender in Research.

Reporting on sex and gender	NA
Population characteristics	NA
Recruitment	NA
Ethics oversight	NA

Note that full information on the approval of the study protocol must also be provided in the manuscript.

Field-specific reporting

Please select the one below that is the best fit for your research. If you are not sure, read the appropriate sections before making your selection.

☐ Life sciences ☒ Behavioural & social sciences ☐ Ecological, evolutionary & environmental sciences

For a reference copy of the document with all sections, see nature.com/documents/nr-reporting-summary-flat.pdf

Behavioural & social sciences study design

All studies must disclose on these points even when the disclosure is negative.

Study description	We assessed changes in healthcare utilization throughout China during the Shanghai outbreak and investigated the relationship between the stringency of population-level mitigation strategies and disruptions in healthcare utilization.
Research sample	All population in Mainland China
Sampling strategy	All population in Mainland China
Data collection	Healthcare service utilization data were from the Center for Health Statistics and Information, National Health Commission of China. Healthcare service utilization volumes in both public and private hospitals at all levels (ie, primary, secondary, tertiary hospitals, and unclassified hospitals) are required to be reported directly on a monthly basis to the provincial-level electronic direct-reporting platforms. Provincial Health Commissions verify and review the data at the primary level, investigate missing reports, and report monthly aggregates to the National Health Commission. The National Health Commission provides technical training and support to staff at the provincial and county level involved in the reporting process, and oversees and checks the quality of data at the secondary level.
Timing	The monthly number of hospital visits (outpatient and emergency department visits) and number of inpatients discharged from hospitals between January 2016 and May 2022 by region (province, municipality, and autonomous region) in mainland China (except Hong Kong and Macau) were obtained.
Data exclusions	NA
Non-participation	NA
Randomization	NA

Reporting for specific materials, systems and methods

We require information from authors about some types of materials, experimental systems and methods used in many studies. Here, indicate whether each material, system or method listed is relevant to your study. If you are not sure if a list item applies to your research, read the appropriate section before selecting a response.

Materials & experimental systems

n/a	Involved in the study
☒	☐ Antibodies
☒	☐ Eukaryotic cell lines
☒	☐ Palaeontology and archaeology
☒	☐ Animals and other organisms
☒	☐ Clinical data
☒	☐ Dual use research of concern

Methods

n/a	Involved in the study
☒	☐ ChIP-seq
☒	☐ Flow cytometry
☒	☐ MRI-based neuroimaging
